# The seed microbiota from an application perspective: an underexplored frontier in plant–microbe interactions

**DOI:** 10.1007/s44297-025-00051-6

**Published:** 2025-06-04

**Authors:** Inês Rebelo Romão, Joana do Carmo Gomes, Daniel Silva, Juan Ignacio Vilchez

**Affiliations:** Instituto de Tecnologia Química E Biológica (ITQB), NOVA, iPlantMicro Lab. Oeiras, Lisbon, Portugal

**Keywords:** Seed microbiota, Microbial inheritance, Seed-borne endophytes, Bioinoculants, Plant–microbe interactions

## Abstract

Seed-associated microbiota represent a critical yet underexplored frontier in plant–microbe interactions, offering unique insights into plant health, resilience, and development. Unlike the soil or rhizosphere microbiome, the seed microbiota is closely tied to plant reproduction, facilitating both vertical and horizontal transmission of microbes. These microbial communities influence key plant processes, including germination, stress tolerance, nutrient acquisition, and pathogen resistance, providing plants with a pre-assembled microbial consortium tailored to their needs. Despite recent advances, significant gaps remain in understanding how seed-associated microbes are acquired, their ecological dynamics, and their functional roles. High-throughput sequencing, metagenomics, and spatial imaging techniques have revealed the diversity and complexity of the seed microbiota, emphasizing their potential for agricultural innovation. This research highlights the importance of these communities in shaping plant resilience and productivity, yet questions about their ecological and evolutionary significance persist. The present review synthesizes current knowledge on the composition, inheritance mechanisms, and functional roles of the seed microbiota. It also explores strategies to harness these microbes for sustainable agriculture, including microbiome engineering and breeding for microbial compatibility. By addressing these gaps, seed microbiota research could revolutionize sustainable agriculture, enhancing crop resilience and reducing reliance on chemical inputs.

## Introduction

In recent years, plant–microbe interactions have garnered significant interest, as researchers have sought sustainable strategies to increase agricultural productivity, reduce chemical inputs, and improve crop resilience [1–3]. Microorganisms associated with plants play crucial roles in nutrient acquisition, stress tolerance, and disease resistance, which in turn influence plant health, yield, and resilience in response to climate change [4–6]. Research has traditionally focused on the soil and rhizosphere microbiomes; however, recent studies have begun to elucidate the critical yet often overlooked role of the seed microbiota in plant development and fitness [7–10]. Over the past decade, there has been a significant surge in interest in this topic, driven by the discovery of its biological relevance in land crops and even in aquatic ecosystems and potential applications [11, 12].

**Fig. 1:**
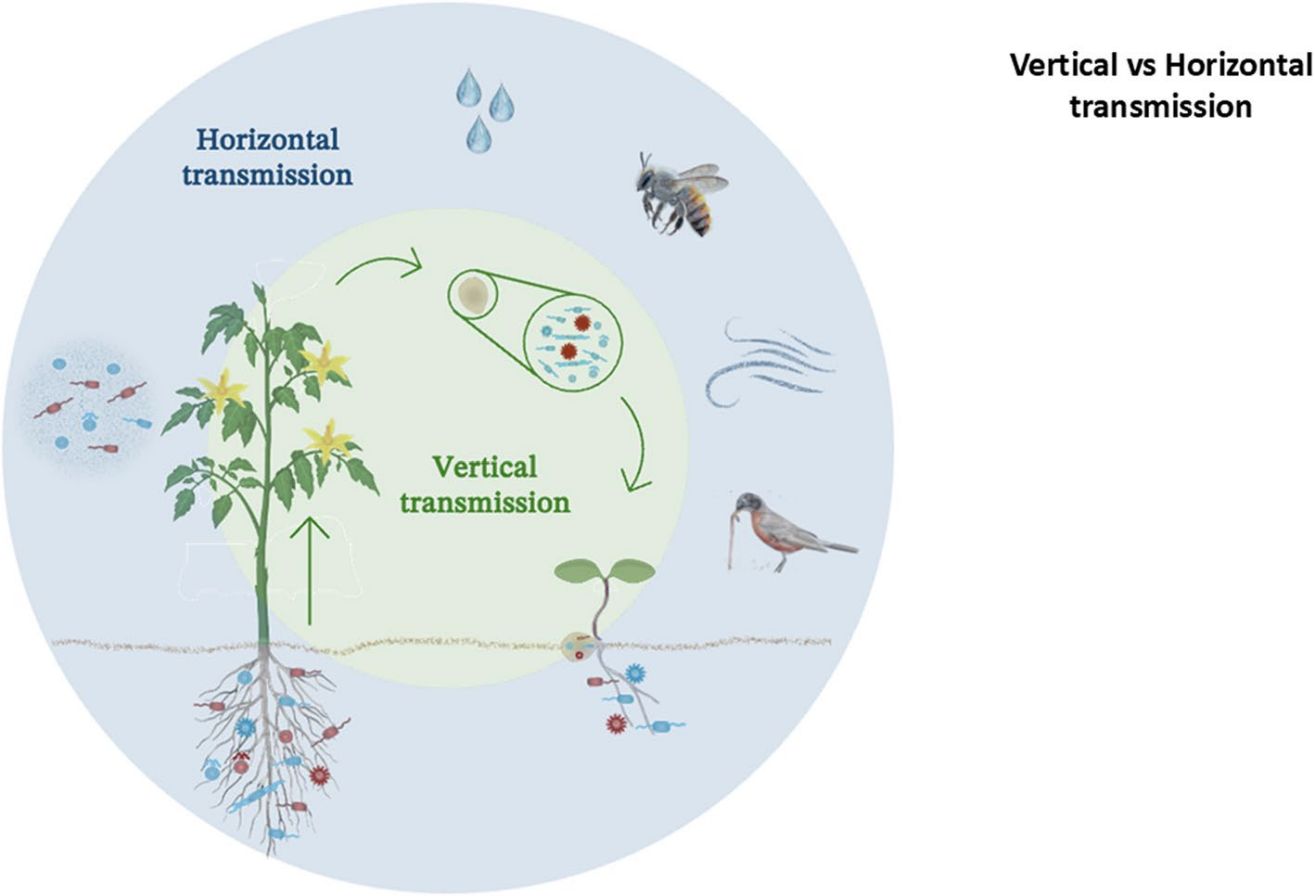
Overview of microbiota transmission pathways in seeds. This diagram integrates the various sources contributing to seed microbiota assembly, illustrating both vertical and horizontal transmission routes. Microbial inheritance can occur vertically through direct parental transfer via seeds, pollen, and maternal tissues, whereas horizontal acquisition arises from environmental sources such as soil, water, air, and interactions with insects or other organisms. These diverse transmission pathways collectively shape the seed microbiome, influencing plant development and adaptation

The term 'seed microbiota' encompasses a diverse community of bacteria, fungi, archaea, and other microorganisms that inhabit the internal and external surfaces of seeds. Unlike soil and root microbiomes, seed-associated microbial communities are unique in that they can be transmitted across generations, providing direct microbial inheritance from parent plants to their offspring [13–18]. This continuity across generations not only ensures a legacy of beneficial interactions but also establishes a foundation upon which subsequent microbiomes, such as those in the rhizosphere and phyllosphere, can develop [19]. Furthermore, this direct microbial inheritance has implications for crop consistency, as it provides plants with a pre-assembled microbiome optimized for early growth and adaptation to the parent plant's environment [20–22]. Microbial inheritance in seeds can occur through two primary pathways: vertical transmission, where microbes are passed from one generation to the next via reproductive tissues; and horizontal transmission, whereby seeds acquire microbes from the external environment, particularly from the soil, during germination [23–26]. Vertical transmission is especially intriguing, as it allows for stable inheritance of specific microbes that may confer selective advantages, such as resistance to pathogens or enhanced nutrient acquisition, especially in stressful or resource-limited environments [14, 27]. In contrast, horizontal transmission enables plants to flexibly recruit beneficial microbes in response to environmental conditions, thereby facilitating their adaptability and resilience in changing ecosystems [24, 28, 29] (Fig. 1).

Plants are not passive hosts in these interactions; they actively select for and shape microbial communities through biochemical and genetic mechanisms. Plant-driven selection processes, which include the release of specific compounds through root exudates or the modulation of immune responses, allow plants to recruit beneficial microbes and inhibit pathogens, thereby creating a tailored microbiome that supports growth and resilience [11, 30]. As such, the microbiome associated with a seed is not only a product of inheritance but also a complex outcome of selective pressures exerted by the host plant, the environmental context, and microbial interactions within the seed microbiome itself.

The functional implications of the seed microbiota extend across plant life stages, from promoting germination and seedling growth to enhancing tolerance to abiotic and biotic stressors. Beneficial microorganisms within seeds can produce phytohormones, solubilize nutrients, and induce systemic resistance to pathogens, all of which contribute to a robust plant phenotype that is better suited for overcoming environmental challenges [7]. Conversely, seeds can also harbor pathogenic microorganisms that pose threats to crop health because these pathogens can establish themselves early in the life cycle of a plant, leading to disease and reduced vigor. Understanding the complex interplay between beneficial and pathogenic microorganisms within the seed microbiota is essential for developing effective strategies to manage plant health and improve agricultural outcomes [8, 15]. Given the central role that the seed microbiota plays in plant fitness and crop productivity, researchers are exploring methods to harness these microbial communities to increase crop resilience and reduce dependency on synthetic fertilizers and pesticides. Approaches such as microbiome engineering and the development of seed bioinoculants offer promising avenues for sustainable agriculture by enhancing the beneficial functions of the seed-associated microbiota and reducing the impact of pathogens [31, 32]. The potential applications of the seed microbiota extend beyond plant health, contributing to broader ecological benefits and advancing sustainable agricultural practices.

This review summarizes the current understanding of the composition of the seed microbiota, microbial inheritance mechanisms, functional implications for plant health, and future applications in agriculture. First, we discuss the diversity and structure of seed-associated microbial communities and examine the factors that shape this unique microbiome. Next, we explored the processes of microbial inheritance, focusing on vertical and horizontal transmission and plant-driven selection mechanisms. Finally, we consider the role of the seed microbiota in promoting plant growth, stress tolerance, and pathogen resistance and discuss future research directions and applications that could harness the seed microbiota to increase agricultural sustainability.

## Composition and diversity of the seed microbiota

The seed microbiota, the microbial community associated with seeds, constitute a critical component of plant–microbe interactions. Over the years, the seed microbiota has demonstrated a distinct composition, particularly in terms of its high concentration of beneficial microorganisms compared with those found in roots and soil. These communities are composed of diverse bacteria, in some instances, archaeal species and fungi, that perform various functions in terms of plant health, influencing seed germination, seedling growth, and resistance to environmental stresses [33]. Recent studies have also begun to explore the presence of yeasts, highlighting their potential role in plant growth promotion and stress resistance [34]. In contrast to other plant-associated microbiomes, the seed microbiota presents unique characteristics that may respond to variable factors, such as the plant's genotype, environmental conditions, and ecosystem interactions [22].

### Core and transient microbiota

Research suggests that the seed microbiota can be categorized into core and transient communities. The core microbiota is stable and consistently presents microbial taxa that reliably colonize seeds across different environments and generations, contributing essential functions to seedling health and development. Transient microbiota are opportunistic or environmental microbes that colonize seeds temporarily—often during dispersal, storage, or germination—and may influence seedling outcomes under specific conditions but do not form part of the long-term, inherited community. The core microbiota is consistently present across diverse plant genotypes and environments and is hypothesized to provide essential functions that benefit the plant [35, 36]. These core microbes include bacterial genera, such as Pseudomonas, Paenibacillus, Bacillus, Pantoea and Streptomyces, as well as fungi, such as Trichoderma and Penicillium, which are commonly found across various plant species and are known for their plant growth-promoting and disease-suppressing capabilities [14, 33].

On the other hand, transient microbiota exhibit greater variability [36, 37] and are often influenced by external factors such as soil type, climate, and agricultural practices [8, 22, 38–41]. These transient microbes may play adaptive roles in specific environments, enabling plants to flexibly respond to changing environmental conditions. Studies have demonstrated that seeds exposed to different soil microbiomes acquire distinct microbial communities, suggesting that the transient component of the seed microbiota can be shaped by environmental factors and that plants may selectively incorporate microbes that are beneficial in specific contexts [22]. However, their presence remains poorly linked to specific events or plant requirements, and it is not possible to determine whether their role in the seed microbiota responds to eventuality or precise recruitment processes.

### Key microbial groups in the seed microbiota

Microbial communities residing within seeds are complex ecosystems dominated by bacteria and fungi, with additional contributions from other microbial groups, such as archaea. These microbial communities perform diverse and critical functions that influence seed health, germination, and development.

Bacteria, including genera such as Pseudomonas, Bacillus, Enterobacter, Micrococcus, and Streptomyces, are among the most prevalent members of the seed microbiota. These bacterial communities play pivotal roles in promoting plant growth and protecting seeds from pathogens. For example, Pseudomonas species are known to produce antifungal compounds that suppress both seed- and soil-borne pathogens, whereas other genera synthesize phytohormones such as auxins, solubilize essential nutrients such as phosphorus, and produce antimicrobial compounds that increase seedling survival and vigor [16, 31, 42–49]. Beneficial Bacillus subtilis strains colonizing melon and wheat seeds produce gibberellins that increase radicle elongation and starch mobilization in the embryo without altering the germination rate [50]. The endophytic Bacillus amyloliquefaciens isolated from rice seeds similarly synthesizes GA in situ, increasing endogenous gibberellin levels to increase seedling vigor and early growth [51]. Moreover, seed priming with B. subtilis triggers the upregulation of IAA-responsive genes and GA biosynthesis pathways, further improving stress tolerance during seedling establishment [52, 53]. Many seed microbiota members express ACC deaminase to cleave 1-aminocyclopropane-1-carboxylate, thereby lowering stress-induced ethylene and indirectly reinforcing ABA-mediated dormancy control under adverse conditions [54, 55]. Conversely, pathogenic Pseudomonas aeruginosa secretes AMB, which triggers DELLA-dependent ABI5 accumulation to arrest germination as a protective response to biotic stress [56]. Volatile organic compounds emitted by diverse seed endophytes modulate auxin and ABA sensitivity, accelerating seedling emergence and enhancing early growth under suboptimal conditions [52, 57]. Together, these examples underscore that the seed microbiota is an active regulator of hormone crosstalk—balancing the gibberellin, auxin, ABA, and ethylene pathways to shape seed development, dormancy depth, and stress resilience [58].

Fungi represent a significant portion of the seed microbiota. Common fungal genera include Trichoderma, Fusarium, Penicillium, and Alternaria spp. These fungi can exert both beneficial and detrimental effects on seeds. Species such as Trichoderma and Penicillium are known for their biocontrol properties, which promote seed and seedling health by outcompeting or antagonizing pathogens. Conversely, some species of Fusarium and Alternaria include pathogenic strains that can compromise seed viability and reduce seedling vigor if they dominate the microbial communities [16, 35, 59–63].

Although bacteria and fungi constitute the majority of the seed microbiota, other microbial groups, including archaea, have been detected, particularly in seeds from extreme environments or unique soil types. Although their roles remain largely unexplored, archaea have been hypothesized to contribute to nutrient cycling and stress tolerance under specific ecological conditions. Moreover, it is also necessary to consider that the complete picture of the seed microbiota remains incomplete, as only a few works have addressed yeast, viruses or other kinds of population-building actors [34, 64–75]. These microorganisms have the potential to elucidate and harness unique functions in agriculture.

### Factors influencing seed microbiota composition

The composition of the seed microbiota is influenced by a range of factors, including plant genotype, environmental conditions, seed developmental stage, and agricultural practices. These factors collectively shape the microbial communities associated with seeds, which in turn can have significant implications for crop resilience and productivity.

The seed microbiome exhibits a wide range of diversity and variability. Consequently, its composition can shift between a flexible group of microbes and a core microbiota [76]. A primary step conducted in assays seeking to gain insights into the diversity of the endophytic microbiota consists of sterilizing the surface of the seeds. There remains a need to assess and apply proper and effective methodologies to obtain the richest culturable endophytic microbiota present without compromising seed viability [77, 78]. The use of non-optimized techniques can lead to thwarted or incomplete results by potentially interfering with both the seeds’ structure and the microbial population’s composition. Despite a wide variation in approach, ethanol, sodium hypochlorite, mercuric chloride and formaldehyde are commonly used as primary means of chemical disinfection and sterilization but can be coupled with other strategies, such as sonication, UV light, or even the use of surfactants such as Triton X-100, Tween 80 and Tween 20 [70, 77, 79].

Plant genotypes play a critical role in determining the structure and diversity of the seed microbiome [36]. Differences among plant species and even among varieties within a species influence seed chemistry, such as exudates and nutrient composition, which directly affects microbial colonization patterns [22]. Certain genotypes have been observed to preferentially associate with beneficial microbes that promote growth and enhance disease resistance. This observation underscores the potential of breeding crops with optimized seed microbiomes to enhance agricultural outcomes [31, 80].

The developmental stage of seeds is another important determinant of their microbiota. Immature seeds often exhibit high microbial diversity, likely due to the active recruitment of microbes from maternal tissues [67, 81, 82]. As seeds mature, selective pressures such as the production of antimicrobial compounds and changes in the seed coat structure act to retain beneficial microbes while excluding potential pathogens [14]. Maternal effects play a pivotal role in this process, as the health and microbial associations of the maternal plant are directly transferred to the seeds. The environmental conditions surrounding the maternal plant, including the soil type, climate, and farming practices, also significantly shape the seed microbiota [20, 39–41, 83]. Diverse soil microbiomes often correlate with more diverse microbial communities in seeds, as they are exposed to soil microbes during their development and after dispersal [24]. Additionally, environmental stressors, such as drought or nutrient limitations, can influence the composition of the seed microbiota by favoring microbes that increase stress tolerance [5, 46].

Agricultural practices and seed treatments further modify the seed microbiota. Practices, such as the application of pesticides and fertilizers, can alter microbial diversity by affecting both pathogens and beneficial microbes. In particular, chemical seed treatments may reduce microbial diversity and potentially affect seedling establishment and growth. Conversely, bioinoculant treatments aim to enhance the seed microbiota by introducing beneficial microbes that promote germination and early-stage growth [31, 32]. Moreover, environmental variables such as temperature, precipitation, and elevation significantly affect the structure of seed‐associated bacterial and fungal communities, as shown for Festuca sinensis seeds on the Qinghai–Tibet Plateau (α‐diversity of bacteria correlated with precipitation; fungi correlated with temperature) [84]. Local “terroir” effects at the farm site can explain more than 12% of the bacterial and nearly 40% of the fungal variance in common bean seed microbiomes, whereas the host genotype often has a negligible impact [39]. In addition, the tillage regime shapes the pool of soil‐borne microbes available for seed recruitment: conservation tillage markedly alters bacterial community composition and assembly processes compared with conventional plowing [85, 86]. Crop rotation likewise reshapes the soil and rhizosphere microbiota, influencing the reservoir of microorganisms that colonize germinating seeds and early seedlings [87, 88]. Finally, edaphic properties such as soil pH, texture, and organic‐matter content at the collection site strongly correlate with seed microbiome assemblages, as demonstrated across 126 fonio millet accessions under variable soil compositions [89].

### Advances in techniques for analyzing the seed microbiota

Recent advancements in microbial sequencing and other omics-based techniques have significantly enhanced our ability to characterize the composition, diversity, and functional roles of the seed microbiota. These advancements have transformed our understanding of the dynamic and complex interactions between seeds and their associated microbial communities [19, 21, 81, 82, 90].

High-throughput sequencing methods, particularly 16S rRNA gene and ITS sequencing, remain fundamental tools for exploring bacterial and fungal diversity within seeds [31, 91–95]. These techniques enable researchers to identify a broad spectrum of microbial taxa and assess their relative abundance, thereby providing a comprehensive view of the composition of the seed microbiota [14]. For example, a study on Zea mays seeds revealed that genera such as Pseudomonas and Bacillus dominate seed bacterial communities, whereas Fusarium and Penicillium are prevalent among fungi [24]. These insights have facilitated the identification of core and transient microbiota, which is crucial for understanding microbial inheritance and recruitment mechanisms. Metagenomics and metatranscriptomics have revolutionized seed microbiota research by enabling the exploration of functional potential and gene expression profiles [31, 91–93, 96]. Metagenomics facilitates the assembly of microbial genomes directly from seed samples, revealing the metabolic pathways employed by microbes to interact with host plants [97, 98]. For example, the functional contributions of genes related to phytohormone biosynthesis, nutrient solubilization, and stress tolerance in seed-associated bacteria to plant health have been elucidated [93]. Metatranscriptomics complements these findings by analyzing active gene expression within the microbial community and elucidating how microbes respond to environmental cues during seed germination or under stress conditions [99–101].

In addition to sequencing-based approaches, advanced imaging techniques such as confocal laser scanning microscopy (CLSM) and fluorescence in situ hybridization (FISH) have provided spatial resolution of the microbial distribution within seeds [102–104]. These techniques allow researchers to distinguish between surface-associated microbes and endophytic communities residing in internal seed tissues. For example, CLSM and FISH were used to detect the presence of Aspergillus sp., Quambalaria sp., Bjerkandera sp., and Staphylococcus sp. in lemon seeds (Citrus limon) [103]. Coupling of FISH and Scanning Electron Microscopy (SEM) was also used to identify niches in the seeds of Cucumis melo [104]. This combination has also been used to observe the distribution patterns and entry points of bacteria and fungi associated with seeds of native alpine plants, revealing unique microbial communities and cross-kingdom microbial interactions [105].

Emerging multi-omics approaches, which integrate data from genomics, transcriptomics, proteomics, and metabolomics, offer unprecedented insights into the functional ecology of the seed microbiota [97, 99, 100]. For example, the integration of metabolomic data with metagenomic profiles has elucidated how the production of specific metabolites by Pseudomonas sp. enhances seedling immunity and growth in barley (Hordeum vulgare) seeds under saline and drought stresses [11, 46, 106]. Proteomics has also been employed to identify microbial proteins that mediate interactions with seed host tissues, such as enzymes involved in cellulose degradation, which facilitate endophyte colonization [31, 107].

Advanced bioinformatics tools are essential for processing and analyzing the vast datasets generated by these techniques. Software platforms, such as QIIME2 and Mothur, are widely used for taxonomic classification and diversity analyses in microbial sequencing studies. Concurrently, machine learning algorithms are increasingly being applied to integrate and interpret multi-omics data, enabling predictive modeling of seed microbiota dynamics and their functional implications [80]. Furthermore, culture-independent approaches such as droplet microfluidics and single-cell genomics are emerging as powerful tools for exploring the functional capabilities of unculturable microbes within seeds. These techniques allow the isolation and analysis of individual microbial cells, circumventing the need for traditional culture methods. For example, single-cell and single-seed genomics have been used to identify novel bacterial species in different crop seeds that contribute to salt tolerance through osmolyte production [81, 90, 108, 109]. As these techniques continue to evolve, they promise to deepen our understanding of intricate interactions within the seed microbiota and their contributions to plant health. The integration of these tools into longitudinal studies and experimental frameworks will help elucidate the mechanisms of microbial inheritance, community assembly, and functional dynamics, paving the way for innovative applications in sustainable agriculture.

## Mechanisms of microbial inheritance

Microorganisms are transmitted to seeds via two primary pathways: vertical transmission, which involves inheritance from the parent plant, and horizontal transmission, in which microbes are acquired from the external environment. These pathways shape the seed microbiota and have significant implications for plant health and resilience. Understanding these mechanisms and their associated examples provides insights into the development and function of the seed microbiota. However, while both vertical (parent-to-seed) and horizontal (environment-to-seed) microbial transmission pathways are recognized, their relative contributions to downstream plant growth traits remain poorly resolved. Further experimental work is needed to delineate how each transmission route uniquely shapes seedling vigor and developmental trajectories.

Vertical transmission ensures that seeds inherit specific microbial communities from their parent plant, often through reproductive tissues such as flowers, ovules, or fruits. For example, Bacillus and Pseudomonas species are commonly inherited from rice (Oryza sativa), where they colonize developing seeds via floral tissues. These bacteria contribute to seedling vigor and provide resistance against pathogens [110–112]. Similarly, in legumes such as Sophora davidii, seed-to-nodule bacteria are vertically transmitted (for example, Sinorhizobium sp.), ensuring that the seeds are primed for nitrogen fixation, even in nutrient-poor soils [9, 37, 113]. This mode of transmission is particularly advantageous in providing plants with a pre-established microbiome tailored to their environment. In wheat (Triticum aestivum), seed microbiota enriched with Bacillus and Arthrobacter have been shown to increase heat and drought tolerance in seedlings by modulating root architecture and producing osmoprotectants [5, 114]. Fungal endophytes also play an important role in vertical transmission. This highlights the role of vertically transmitted fungi in promoting plant resilience to abiotic stress [16]. Another notable example is the colonization of tomato (Solanum lycopersicum) seeds by Streptomyces species, which leads to different plant health traits [115]. These bacteria exhibit antimicrobial properties that protect seedlings from pathogens such as Fusarium sp., ensuring healthy early development [61, 116, 117]. Vertical transmission provides stability across plant generations by consistently transferring beneficial microbes. However, this approach is inherently limited to microbial communities present in the parent plant, which may constrain the diversity of the inherited microbiome. Horizontal transmission complements this process, allowing seeds to acquire microbes from their surroundings.

Horizontal transmission is more dynamic and occurs when seeds interact with external microbial sources, such as soil, water, or nearby plants, during or after dispersal. For example, maize (Zea mays) seeds recruit Azospirillum brasilense, a nitrogen-fixing bacterium, from soil during germination. This interaction enhances root development and nitrogen uptake, particularly in soils with low fertility [119–122]. Similarly, barley seeds often acquire Trichoderma harzianum from fungus-rich soils [123]. This fungus produces phytohormones such as gibberellins and protects seedlings against root pathogens, demonstrating the adaptability and functional benefits of horizontally acquired microbes. Several crops present a consistent horizontally transmitted community that is related to soil-imbibition processes, which are relevant for preparing seedlings for harsh conditions [24, 124]. In this sense, it has been reported that a variable-in-presence Pantoea agglomerans strain establishes itself as part of the rhizosphere microbiota, where it produces siderophores that inhibit pathogens such as Alternaria alternata [11, 44]. The flexibility of horizontal transmission enables plants to recruit microbes that are adapted to specific environmental conditions, thereby increasing their adaptability and resilience. Horizontal transmission plays a crucial role, even in extreme environments. For example, seeds from the metal hyperaccumulators Brassicales and Asterales or from species such as Noccaea carulescens presented a limited stable microbiota. This microbiota, for example, the Gammaproteobacteria, Actinobacteria, and Bacteroidia phyla acquired from metal-rich soils, may be particularly adaptable to hyperaccumulation strategies and promote plant growth in harsh environments [125, 126]. These findings demonstrate that seeds can selectively incorporate microbes to facilitate their adaptation to challenging ecological contexts.

Regardless of the transmission mode, despite the high variability and relatively low diversity, there is stable and consistent transmission of part of the endophytic microbiome, suggesting that their assembly or recruitment is not a result of stochasticity. Notably, in a multigenerational context, the transmission of the seed microbiome can surpass different abiotic effects and conditions, even under environmental stress [127]. The interplay between vertical and horizontal transmission provides seeds with a balance between stability and adaptability. Although vertical transmission ensures the inheritance of core microbiota optimized for the parent plant environment, horizontal transmission allows seeds to dynamically recruit additional microbes on the basis of their immediate surroundings. Together, these processes establish diverse functional microbiomes that support seed germination, seedling establishment, and overall plant fitness. Elucidating the mechanisms and outcomes of these transmission pathways offers valuable insights into harnessing seed microbiota for sustainable agricultural practices (Table 1).

**Table 1:** Resumed comparison of vertical and horizontal transmission [37, 128–130]

Feature	Vertical Transmission	Horizontal Transmission
Source of microbes	Parent plant tissues	External environment (soil, water, other plants)
Microbial diversity	Limited to inherited species	Highly diverse, context-dependent
Timing	During seed development	During dispersal, imbibition, or germination
Stability	High (stable inheritance across generations)	Variable (dependent on environmental conditions)
Examples	*Bacillus*, *Pseudomonas*, or *Rhizobium* in rice, legumes	*Azospirillum*, *Trichoderma*, *Ppseudomonas*, or *Pantoea* in maize, barley

### Vertical transmission

**Fig. 2:**
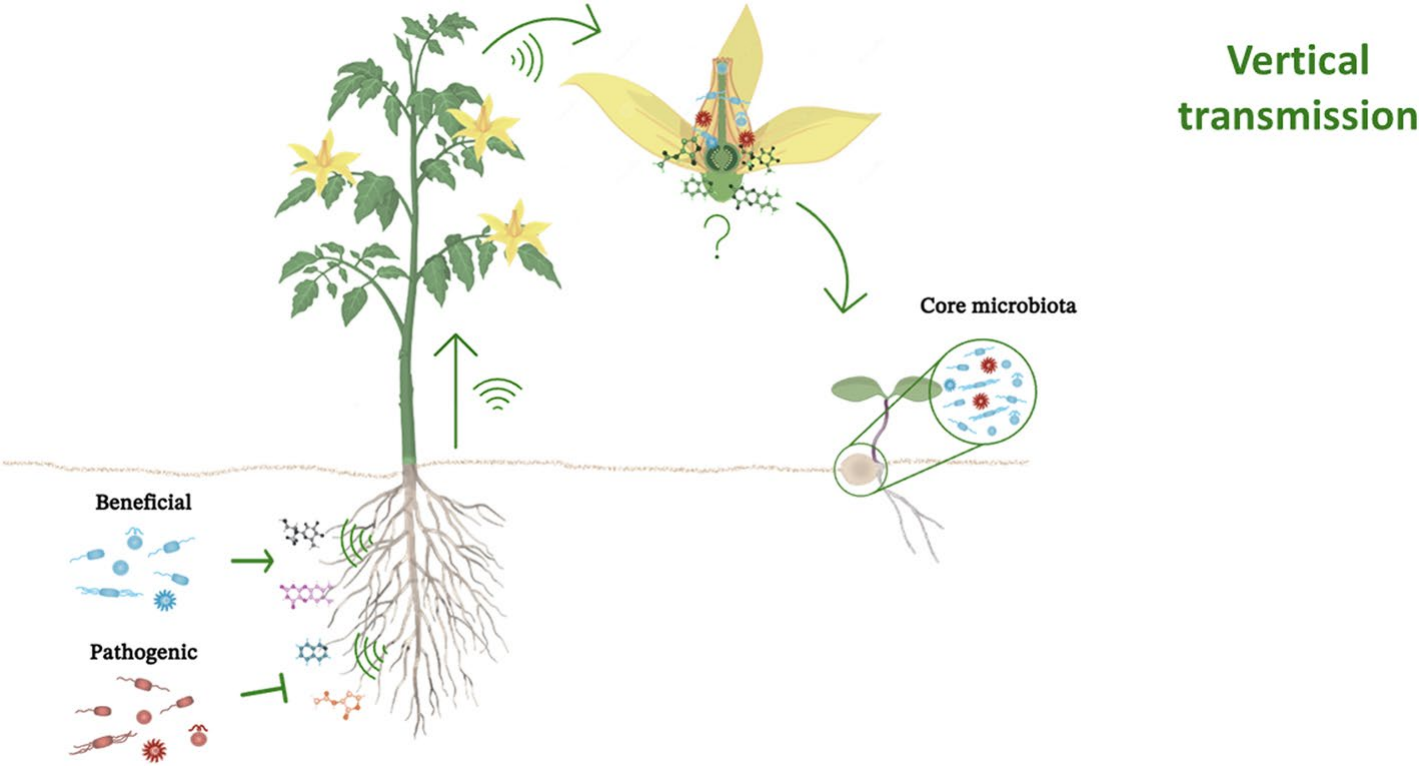
Schematic representation of the vertical transmission mechanisms of the seed-associated microbiota. The diagram outlines a sequential process wherein a) Root exudate signaling actively recruits beneficial soil microorganisms; b) Plant-derived systemic signals facilitate the directed migration of these microbes toward the reproductive organs; c) At the floral level, selective chemical barriers modulate microbial entry by promoting beneficial taxa while excluding potential pathogens; and d) The resultant core microbiota is integrated into the developing seeds, thereby ensuring the transmission of advantageous microbial consortia to the next generation

Vertical transmission is a crucial mechanism that facilitates the direct transfer of microbial communities from parent plants to their seeds, thereby ensuring the perpetuation of beneficial microbes across generations. This inheritance pathway is mediated by plant reproductive tissues such as flowers, ovules, and pollen, where specific microbial taxa colonize and integrate into developing seeds (Fig. 2). These microbes constitute the "core microbiota" essential for seed germination, early seedling development, and pathogen resistance [10, 73]. The reproductive organs of maternal plants function as critical sites for microbial colonization. For example, endophytic bacteria such as Bacillus subtilis and Pseudomonas fluorescens are consistently identified within floral tissues and are vertically transmitted into seeds across various crops, including wheat and rice. These bacteria are known for their growth-promoting and pathogen-suppressing properties [11, 118, 130, 131]. Studies on maize (Zea mays) have evaluated the role of Enterobacter sp. in the colonization of floral organs and ovules, facilitating the transfer of their ability to subsequent generations [41, 132].

Over the years, studies have been able to prove vertical transmission, although the mechanisms that allow vertical transmission to occur are still unknown [133–135]. Several biological mechanisms are hypothesized to support vertical transmission. For example, research on Arabidopsis thaliana has elucidated the role of plant immune modulation in seed-transmitted Cucumber mosaic virus [64]. It is then hypothesized that host plants may employ chemical signaling pathways to attract and sustain or even modulate and repel specific microbes in their reproductive tissues [21, 23, 76, 83, 118, 136].

Vertical transmission fidelity confers evolutionary advantages by establishing a stable, inherited microbiome that co-evolves with the plant host. In rice, vertically transmitted endophytes have been demonstrated to increase seedling tolerance to abiotic stresses such as drought and salinity. The bacterial genera Streptomyces and Bacillus play crucial roles in the production of osmoprotectants and stress-modulating compounds, thereby ensuring seedling survival under challenging environmental conditions [5, 46, 65, 137, 138]. Similar findings in maize and wheat demonstrate that vertically inherited microbiota support nutrient acquisition, stress tolerance and pathogen resistance during the early developmental stages [5, 82, 114, 122, 131, 132, 139, 140]. Particularly in wheat, the culturable diversity of next-generation seeds was greater in the heat-sensitive variety than in the heat-tolerant variety. In the latter, the bacterial genus Bacillus was the most predominant, exhibiting both heat tolerance and plant growth-promoting (PGP) activities [114]. As mentioned previously, studies conducted on Sophora davidii support the results presented above, demonstrating the transmission of the core microbiota from seeds to nodules, including rhizobial endophytes [37].

However, vertical transmission of microbes is not without risk. Pathogens can exploit this pathway to persist across generations, often leading to systemic infection. For example, Fusarium and Alternaria have been documented in maize and wheat seeds as vertically inherited pathogens capable of reducing seed germination rates and compromising crop yield [41, 62, 117, 141–143]. Recent studies have underscored the critical influence of microbial signals on seed germination and early plant development. Pathogenic bacteria such as Pseudomonas aeruginosa can actively inhibit germination through the secretion of bioactive compounds such as AMB, which modulate hormonal pathways independently of gibberellins [144]. Similarly, rhizobacteria expressing ACC deaminase, such as Pseudomonas putida KT2440, suppress ethylene production and reinforce ABA signaling to arrest germination in multiple plant species [145, 146]. These findings highlight that microbial communities surrounding seeds are not passive residents but active regulators of hormonal crosstalk, stress perception, and germination timing. This emerging understanding emphasizes the relevance of the seed microbiota in modulating plant fitness under both biotic and abiotic conditions. Despite this challenge, host plants have evolved sophisticated immune responses to mitigate pathogen proliferation. The connection between plant genetic control and microbial selection further underscores the complexity of vertical transmission. Breeding programs targeting enhanced seed microbiota are currently investigating their potential to selectively promote beneficial endophytes while minimizing pathogen inheritance. For example, studies on soybeans (Glycine max) have demonstrated that plants bred for high-nitrogen fixation also exhibit enriched populations of beneficial seed microbiota, highlighting the interconnected roles of genetic and microbial inheritance. Ongoing research on the mechanisms governing vertical transmission presents promising opportunities for harnessing this natural inheritance process to increase crop resilience. Elucidating the selective pressures exerted by plants on seed-associated microbes coupled with advances in microbiome engineering may facilitate the development of next-generation crops with optimized microbiota for sustainable agriculture.

### Horizontal transmission

**Fig.3:**
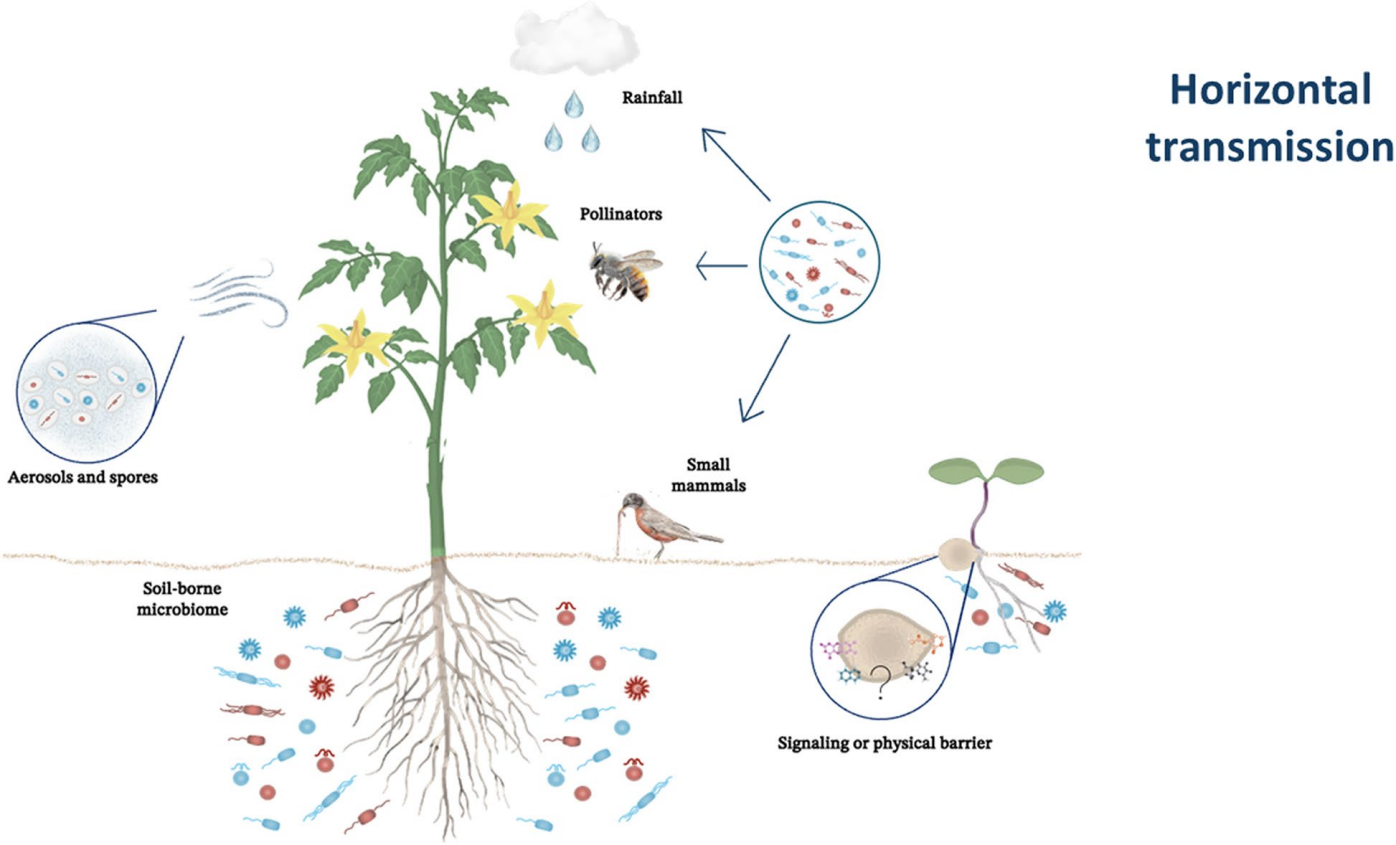
Schematic representation of horizontal transmission pathways shaping the seed microbiome. The diagram integrates multiple environmental sources contributing to microbial acquisition: the bulk soil at the base acts as a reservoir of diverse microorganisms; aerosols and wind-dispersed spores (left panel) facilitate airborne transport; and rainfall, together with biotic vectors such as pollinators and small mammals (right panel), promotes microbial deposition on floral tissues and early germinating seeds. Additionally, a putative selective barrier at the outer seed coat is indicated, suggesting a role in modulating microbial entry

Horizontal transmission plays a pivotal role in shaping the seed microbiota by facilitating the recruitment of beneficial microbes from diverse environmental sources. These commonly can come either from the soil or flowers, which are located on petals, stamens, and pistils, often arriving via pollinators, wind, or water, as shown in Fig. 3 [28, 29, 147–149]. This transmission pathway is essential for enhancing microbial diversity within seeds and equipping plants with the adaptability required to thrive under varying ecological conditions. Unlike vertically inherited microbes, horizontally acquired microbes offer seeds a dynamic array of functionalities that enable plants to respond to local environmental pressures, ranging from nutrient limitations to biotic and abiotic stresses.

Soil serves as a major reservoir for microbial recruitment, with studies consistently demonstrating its influence on seed microbiota composition. For example, wheat (Triticum aestivum) seeds acquire beneficial strains from the surrounding soil, which are known for their ability to promote seedling growth and suppress pathogenic fungi [41, 123, 139, 150–153]. Similarly, soil conditions, such as pH, moisture, and nutrient availability, directly influence the recruitment of microbes, creating an environmentally driven selection process that contributes to shaping the seed microbiota [24, 26, 41, 153]. Environmental dispersal mechanisms also play a significant role in horizontal microbial transmission. Wind can transport bacterial aerosols and fungal spores, enabling their deposition onto seeds during maturation and dispersal. Species such as Cladosporium and Penicillium, which are commonly dispersed through air currents, often opportunistically colonize seeds, particularly in regions with high humidity [97, 139, 154]. Similarly, rainfall functions as a vehicle for microbial deposition, particularly in open-flowering plants, where water droplets transfer microbes from the atmosphere or surrounding vegetation to the seed surface [38, 155]. These interactions introduce diverse microbial taxa into seeds, which can subsequently influence germination success and seedling development.

Biotic interactions further increase the horizontal transfer of microbes to seeds. Pollinators, including bees, butterflies, and beetles, contribute significantly by depositing microbes onto floral structures, which are subsequently transmitted to developing seeds. Research has identified bacterial genera such as Pseudomonas and Enterobacter in the flowers and seeds of multiple crop species, highlighting their roles in pathogen suppression and nutrient mobilization [156–159]. Furthermore, animals that disperse seeds, such as birds and small mammals, may play a role in introducing unique microbial communities to seeds, either during digestion or through external transport. For example, the seeds of frugivorous birds that pass through their digestive tracts enrich their seed microbiota with strains capable of improving seedling establishment in nutrient-depleted soils, whereas those of hummingbirds are cross-sharing microbiota, influencing the nectar and flower microbiomes, especially bacteria and yeast [74, 160–164].

Notably, seeds are not entirely passive recipients of microbial colonization but actively regulate horizontal transmission through physical barriers and signaling mechanisms. During seed formation, the seed coat accumulates hydrophobic waxes, phenolic compounds, and polysaccharides that serve as selective filters, recruiting beneficial microbes while excluding potential pathogens. For example, phenolics in legume seed coats attract nitrogen-fixing Rhizobium species while deterring fewer desirable taxa [165, 166]. In addition, seed exudates released during germination contain metabolites that influence microbial recruitment. Flavonoids and sugars, in genera such as Rhizobium and Bacillus, act as chemoattractants for beneficial microbes, promoting symbiotic associations that enhance seedling development [30, 167]. Nevertheless, the microbiota that is horizontally transmitted also consists of a share of microorganisms that are in bulk in the soil. Owing to their high presence in the soil, the chance of seed colonization increases exponentially [23]. Agricultural practices significantly shape the horizontal transmission of microbes. For example, bioinoculants are increasingly utilized to augment the seed microbiota by introducing beneficial strains during planting. Common bioinoculants, including Trichoderma and Pseudomonas, increase plant resistance to pathogens and improve nutrient uptake [11, 31]. Such practices not only promote the establishment of beneficial microbes but also mitigate the impact of intensive farming practices that reduce microbial diversity in agricultural soils [11].

Emerging research has highlighted that horizontal transmission mechanisms are deeply integrated into plant-driven selection processes. Molecular studies have revealed that the compounds in the seed coat and seed exudates modulate microbial colonization by selectively enriching taxa with specific functionalities. For example, halophyte seeds exposed to saline soils preferentially recruit salt-tolerant microbes, which improve the osmotic balance under high salinity stress [49]. Moreover, laboratory-based biopriming techniques, which harness these natural mechanisms, have been developed to increase seed performance [168, 169]. These findings reinforce the adaptive importance of horizontal transmission in plants equipped with locally tailored microbiomes that increase their fitness and resilience. The ecological implications of horizontal microbial transmission are profound because they allow plants to adapt flexibly to dynamic environmental conditions by recruiting microbes with context-specific benefits. As research continues to elucidate the complex mechanisms underlying this process, horizontal transmission represents a critical interface between plant biology and microbial ecology, offering insights into how seeds dynamically assemble their microbiomes to support plant health and productivity.

### Plant-driven selection of seed microbiota

Plants actively shape the microbial communities associated with their seeds through various biochemical, genetic, and structural mechanisms. These processes enable plants to assemble microbiomes that support plant growth, stress resilience, and disease resistance. Seed microbiota selection involves complex interactions among host traits, environmental factors, and microbial dynamics, with implications for plant health across generations [170].

A prominent mechanism by which plants influence the seed microbiota is through the production of secondary metabolites and signaling compounds. These molecules selectively recruit beneficial microbes and deter pathogens. For example, phenolic compounds and flavonoids secreted by plants attract beneficial taxa that are known for their biocontrol and growth-promoting properties. Several studies on seeds have demonstrated that specific secondary metabolites shape the seed microbial community by influencing microbial colonization patterns [25, 129, 139, 171]. Seed structural features also play crucial roles in microbial selection. The seed coat, which is composed of a complex matrix of polysaccharides, proteins, and phenolic compounds, functions as a selective barrier. Research on maize and soybean has shown that seed coat composition can influence the abundance and diversity of associated microbial taxa, favoring beneficial microbes [97, 172, 173].

Host immune responses are another critical factor in shaping the seed microbiota. Plants employ immune signaling pathways, such as those mediated by salicylic acid and jasmonic acid, to selectively suppress pathogens and recruit beneficial microbes. For example, salicylic acid signaling in rice has been linked to the exclusion of harmful bacteria while supporting the colonization of Bacillus plant growth-promoting bacteria and may also play a role in the assembly of the seed microbiota [14, 21, 31, 39, 48, 76, 83]. Environmental factors also modulate the influence of plants on the seed microbiota [25, 48, 82, 90, 95]. Hence, maternal plant health, stress resilience and the surrounding soil microbiome during seed development should affect the composition of the microbial community [23, 24, 26, 39, 40, 76, 82, 94, 134, 174]. Research on wild Panax or quinoa has shown that soil microbial diversity and maternal plant conditions can drive variations in the seed microbiota, emphasizing the interplay between environmental and host factors [17, 35, 36, 175]. Although the mechanisms underlying the plant-driven selection of seed microbiota have not been fully elucidated, advances in omics technologies have provided insights into these complex interactions. Metagenomic and metabolomic studies have revealed distinct microbial assemblages in the seeds of different genotypes and different degrees of domestication, highlighting the influence of host genetic traits [18, 39, 41, 76, 133, 175–177]. This growing body of research underscores the potential of leveraging plant-driven selection processes to improve agricultural sustainability. By breeding crops with traits that favor beneficial microbial associations or developing microbial consortia that complement plant-driven selection, researchers can optimize the seed microbiota to increase resilience and productivity [83, 178, 179].

### Microbial interactions and co-evolution with the host

The intricate interplay between plants and their associated microbes reflects the extensive history of co-evolution that has shaped the seed microbiota. This relationship influences microbial inheritance within seeds and plays a crucial role in plant fitness [14, 132, 133, 175]. Symbiotic relationships, such as those between legumes and nitrogen-fixing bacteria or endophytic fungi and their hosts, have evolved over millions of years, creating highly specialized interactions that are often specific to plant species or genotypes [180, 181]. These kinds of co-evolutionary processes may establish the foundation for microbial inheritance, enabling plants to carry beneficial microbial communities through seeds and adapt to diverse ecological conditions [10, 30, 71, 132, 133].

In seed microbiomes, this coevolution is evident in the selective retention of beneficial microbes that increase plant health [165]. For example, nitrogen-fixing species have been detected in Brassica seeds, underscoring the evolutionary importance of maintaining symbiotic relationships that ensure access to nitrogen, a critical nutrient that is often limited in natural environments [40, 150]. Similarly, endophytic fungal communities have been found to colonize seeds with a bacteria-dependent component, where they provide early protection against pathogens and promote nutrient uptake, benefiting plants during germination and early growth [60, 63]. The selective pressure exerted by plants further drives these associations. For example, signaling molecules, such as strigolactones, flavonoids and some organic acids, which are very specifically produced molecules, mediate interactions with arbuscular mycorrhizal fungi and nitrogen-fixing bacteria, but they also acting as anti-pathogen compounds [182–184]. Although these signals are traditionally associated with root interactions, they are firm candidates that may also influence seed-microbiota assembling, thereby shaping the microbial community inherited by the next generation [183–186]. These biochemical interactions associated with the recruitment and assembly of the seed microbiota remain poorly understood, but the early results seem to indicate a high degree of specificity. In addition, microbial interactions within the seed microbiome, including cross-feeding and quorum sensing, have been recognized as pivotal mechanisms for stabilizing these communities and enhancing their collective functionality [9, 76, 110, 174, 187–190].

Furthermore, advancements in functional genomics have elucidated the genetic basis of plant–microbe co-evolution, particularly in terms of the impact of domestication processes on the associated microbiota [41, 176, 190]. These co-evolutionary dynamics have substantial potential for crop improvement. By leveraging the principles of plant–microbe co-evolution, researchers have designed microbial consortia that emulate natural processes but also enhance seed microbiota functionality [32, 79]. Moreover, selective breeding programs targeting plants that naturally favor beneficial microbiota offer promising avenues for enhancing agricultural resilience and productivity in the face of climate change [10, 48, 99, 179, 180]. The co- evolutionary perspective emphasizes the dynamic and adaptive nature of the seed microbiota, which is shaped by millions of years of plant–microbe interactions. By elucidating the genetic, biochemical, and ecological foundations of these interactions, researchers can harness the power of coevolution to develop innovative and sustainable agricultural solutions.

### Functional implications of the seed microbiota for plants

The seed microbiota provides several essential functions that contribute to plant fitness, growth, and adaptability. Through colonization of the seed at its earliest developmental stage, microorganisms influence the commencement of germination, thereby shaping the initial establishment and development of the plant [16, 59, 160, 191]. Research has demonstrated that the seed microbiota enhances nutrient uptake, promotes growth through hormonal pathways, confers protection against pathogens, and improves plant resilience to environmental stresses [11, 23, 110, 192]. These functions are particularly valuable in sustainable agriculture, wherein optimization of the seed microbiome can result in healthier crops with reduced inputs and enhanced resilience under suboptimal growth conditions (Table 2).

**Table 2:** Examples of the effects of the seed microbiome as a treatment for abiotic and biotic stresses

Strain	Plant	Effect In Plant	Refs
*Bacillus sp.*	Soybean	Biocontrol effect against *Diaporthe eres* and improvement of germination	[193]
*Herbaspirillum sp.*	Wheat	Promotion of root growth by alteration of the tryptophan metabolism	[194]
*Pseudomonas sp.*	Maize	Demonstrated the ability to promote growth under drought conditions	[104]
	Barley	Improved growth, metal acquisition, photosynthesis, and oxidative stress tolerance in drought-stressed	[46]
	Soybean	Antagonistic activity against seed-borne pathogens	[196]
*Phomopsis sp.*	Soybean	Antagonistic activity against seed-borne pathogens	[195]
*Microbacterium sp.*	Rice	Promotes vegetative growth under saline stress	[196]
*Flavobacterium sp.*	Rice	Promotes vegetative growth under saline stress	[196]
*Paenibacillus sp.*	Maize	Antagonistic activity against fungal pathogens	[197]
	Lentil	Demonstrated the ability to promote growth under drought conditions	[198]
	Tobacco	Antagonistic activity against *Ralstonia solanacearum*	[188]
	Mongolian milkvetch	Enhanced seed germination and antagonistic activity against *Fusarium solani*	[108]
*Burkholderia sp.*	Rice	Antagonistic activity against fungal pathogens (*Marasmius graminum*, *Rhizoctonia solani*)	[199]
	Maize	Promotion of phosphorus acquisition by the host plant by colonizing the rhizosphere	[48]
*Curtobacterium sp.*	Wheat	Demonstrated the ability to promote growth under drought conditions	[5]
*Arthrobacter sp.*	Wheat	Demonstrated the ability to promote growth under drought conditions	[5]
*Pantoea sp.*	Barley	Improved growth, metal acquisition, photosynthesis, and oxidative stress tolerance in drought-stressed barley	[46]
	Rice	Antagonistic activity against fungal pathogens (*Marasmius graminum* and *Rhizoctonia solani*)	[199]
*Pichia kudriavzevii*	Black gram	Improved the seed germination, physiological parameters and yield	[75]
*Issatchenkia terricola*	Black gram	Improved the seed germination, physiological parameters and yield	[75]
*Stenotrophomonas sp.*	Lentil	Demonstrated the ability to promote growth under drought conditions	[198]
	Maize		[104]
*Enterobacter sp.*	Lentil	Demonstrated the ability to promote growth under drought conditions	[198]

### Enhanced nutrient acquisition and phytohormone production

Seed-associated microorganisms are crucial contributors to nutrient acquisition, facilitating the accessibility of essential elements such as nitrogen, phosphorus, and iron to seedlings [16, 76, 110, 116, 200]. These microorganisms frequently interact during early seed development and establish a preexisting system for nutrient mobilization. For example, nitrogen cycle-related genera of bacteria, such as Sphingomonas sp., Chryseobacterium sp., Methylobacterium sp., Paenibacillus sp., Pantoea sp., and Pseudomonas sp., were detected in soybean seeds. These compounds directly contribute to plant nitrogen requirements [193, 201]. In addition to nitrogen, the seed microbiota plays a pivotal role in phosphorus solubilization, which is essential for low-phosphorus soils. Seedborne Sphingomonas sp., Pseudomonas sp., Pantoea sp., Bacillus subtilis, Microbacterium sp., and Curtobacterium oceanosedimentum isolates have been shown to solubilize inorganic phosphate and enhance seedling growth in rice by improving phosphorus bioavailability but also produce siderophores and even fix nitrogen [202]. We also found that some strains of Burkholderia sp. have been linked to phosphorus solubilization in maize seedlings [48]. Similarly, in chickpea seeds, Enterobacter sp., Bacillus sp., Pseudomonas sp., Staphylococcus sp., and Pantoea sp. have been reported to be inherited symbionts that play a significant role in early phosphorous and potassium solubilization, incorporating ammonia production [116]. Most of these cases also report strains that can solubilize other nutrients, such as Ca, Zn, Mn or Si, which increases nutrient availability and may increase the likelihood of survival in emerging seedlings.

Moreover, the seed microbiota frequently synthesizes phytohormones that directly impact plant growth and development. Auxin production, particularly that of indole-3-acetic acid (IAA), and the control of ethylene by 1-aminocyclopropane-1-carboxylate (ACC) deaminase are characteristic features of numerous seed-associated bacteria [202]. Strains of Micrococcus yunnanensis, Micrococcus luteus, Enterobacter soli, Leclercia adecarboxylata, Pantoea dispersa, and Staphylococcus epidermidis, which were isolated from rice seeds, produced high amounts of IAA. They are used as inoculants, causing root elongation and lateral root development, consequently improving water and nutrient uptake in rice seedlings [203]. Similarly, several strains of Bacillus sp. and Paenabacillus sp. isolated from Cucurbitaceae family plants presented high growth-promoting activities linked to IAA and ACC deaminase production, as well as other nutrient-related traits [200]. In addition to being reported as phytohormone producers, several fungi isolated from Senna alata seeds, namely, Fusarium sp., produced high levels of IAA and promoted plant growth when they were used as bioinoculants [204].

### Pathogen suppression and disease resistance

The seed microbiota plays a pivotal role in protecting plants from pathogens by employing mechanisms such as resource competition, the production of antimicrobial compounds, and the induction of systemic resistance [188, 192, 205]. For example, Bacillus velezensis, which is isolated from peanut seeds, produces bacillomycin A, surfactin A and fengycin A with antifungal properties that effectively inhibit Sclerotium rolfsii, a common seedling pathogen [206]. Interestingly, a strain of Paenibacillus sp. isolated from maize demonstrated a wide range of antagonistic effects and was able to control pathogens such as Fusarium graminearum, Bipolaris maydis, Bipolaris sorokiniana, Cochliobolus heterostrophus, Aspergillus aculeatus, Phomopsis chimonanthi and Verticillium dahlia, delineating a great protection barrier for seedlings in their more delicate developmental moment [197]. This strategy is enhanced by the capacity of beneficial microbes to occupy seed and root niches effectively, thus limiting the potential for pathogenic colonization [113, 188, 192, 205, 207]. In this sense, some fungal strains isolated from moso bamboo seeds, namely, Cladosporium sp., Curvularia sp., Penicillium sp., and Shiraia sp., have also been reported as antimicrobial producers and pathogen controllers, competing for the seed niche with other fungi that were identified as pathogens. For the first time, the Shiraia sp. strain was identified as a hypocrellin A producer, an antimicrobial compound for many bacterial and fungal pathogens [208]. On the other hand, several Trichoderma sp. strains isolated from bean seeds prime plant growth and development, thereby enabling plants to respond more effectively to Rhizoctonia solani pathogen attacks [209]. Similarly, Bacillus species, the core microbiota found in resistant maize varieties, were shown to suppress corn stalk rot (Fusarium graminearum) by inducing the production of berberine by the host [131]. As a final example, a strain of Pseudomonas koreensis isolated from soybeans was able to confer resistance against all the seed-borne pathogens, such as Cercospora kikuchii, Cercospora sojina, Diaporthe eres, and Septoria glycines, while Pseudomonas syringae pv. tabaci and Xanthomonas axonopodis pv. glycines via the production of antimicrobial metabolites and volatiles [195].

However, in addition to beneficial microorganisms, seeds can also harbor and spread pathogenic microbes. These seed-borne pathogens colonize seeds via their presence in the environment or through vertical transmission, which affects mainly seed germination and crop yield [62, 142, 210]. In addition, seeds are effective microbial vectors, and the transmission of seed-borne pathogens into other geographical areas might impose a large risk for agriculture [111, 113, 141, 143, 170, 210]. The development of systems to track and identify the core quarantine pathogens of barley has already occurred and is the starting point of an emerging topic [106]. Several cases reported that a beneficial microbiota assembly in seeds was enough to cope with these infection-causing pathogens (niche competition), but they still posed a risk of dissemination and disease spread [188, 192, 205].

### Increased stress tolerance to abiotic stresses

The seed microbiota also plays a role in enhancing plant resilience to environmental stressors such as drought, salinity, and extreme temperatures. With respect to drought tolerance, many cases in which the seed microbiota from maize was able to protect growth and production under water scarcity conditions were identified. For example, strains of Priestia sp., Burkholderia sp., Psuedomonas koreensis, Pseudomonas fulva, and Stenotrophomonas sp. were demonstrated to be able to produce ACC deaminase, auxins and biofilms, even under drought conditions. All these traits improved water availability and retention in the rhizosphere and reduced water stress in the seedlings, which, even in the field test, improved development and production under stress conditions [104]. In addition to the use of the maize seed microbiota, many isolated strains produced notable amounts of antioxidants, and their use as bioinoculants improved vegetative growth and plant responses to stress, increasing proline, sugar, and protein contents under normal and drought conditions [211]. In another study, strains of Paenibacillus sp. and Stenotrophomonas sp. and strains of Enterobacter hormaechei isolated from Medicago sativa and Bituminaria bituminosa also presented similar protective traits and were used to improve drought tolerance and development in sensitive lentils (Lens culinaris) [198]. Other works using seeds isolated from wheat Curtobacterium flaccumfaciens and Arthrobacter sp. have shown that increasing microbial-associated communities in the rhizosphere enhances water scarcity tolerance [5]. A Kosakonia cowanii strain isolated from lettuce (Lactuca serriola) with high production of exopolysaccharides under stress conditions also protected seedlings, suggesting the importance of protective structure production under such conditions in plant protection [212].

These protectants also play a pivotal role in salt stress. Thus, Rummeliibacillus stabekisii, Peribacillus frigoritolerans and Pseudomonas protegens isolated from tomato seeds were able to increase the osmotic stress resistance of tomato plants through the production of protectants [115]. Metagenomic analyses have shown that the seed microbial diversity in alkali- and saline-tolerant rice is unique and is related to the saline tolerance of such varieties [213–215]. The extreme temperatures also shape and condition the microbial communities in seeds, as was reported in different heat-responsive genotypes of wheat, where Bacillus was dominant in most heat-tolerant cases [45, 136]. Moreover, a Pseudomonas aeruginosa strain isolated from wheat seeds was reported to produce heat-shock proteins that stabilize plant enzymes under high-temperature conditions, thereby improving seedling survival in heat-stressed environments [114]. Similarly, cold treatments modify the A. thaliana seed microbiota by modulating the composition of lipids and other metabolites [216]. In the context of other abiotic stresses, such as heavy metal contamination, the seed microbiota is clearly relevant in terms of strain and whole population composition to increase the performance of hyperaccumulating plants [125, 126]. With more research on the seed microbiota, other strains and populations may emerge as stress-related inoculants or systemic protectants of the host plants.

### Implications for sustainable agriculture

The functional benefits of the seed microbiota make it a significant tool for sustainable agriculture [8]. By reducing their reliance on chemical fertilizers and pesticides, seed-associated microbes offer natural and environmentally sustainable alternatives to conventional farming practices. Here, the ‘intimacy’ level between the plant and seed microbiota must be considered, as this could constitute a differential trait when facing inoculant efficiency, as this relationship may lead to preadaptation in the plant‒microbe interaction [8, 25, 26]. Moreover, future agricultural strategies may include breeding crops with enhanced compatibility with beneficial seed microbiota or developing bioinoculants and synthetic communities that integrate multiple functional traits tailored to specific crops and environments [32, 79, 98, 147, 179, 180]. By harnessing the natural capabilities of the seed microbiota, agriculture can achieve sustainable intensification while maintaining environmental health and resilience.

## Applications of the seed microbiota and future directions: Development of seed microbiota bioinoculants, breeding and synthetic approaches

The potential to utilize the seed microbiota in agriculture has garnered significant attention as agricultural practitioners, researchers, and policymakers seek methods to increase the sustainability and resilience of crop production [113, 172]. Seed-associated microorganisms are fundamental to plant early health and growth, making them promising candidates for reducing chemical inputs, increasing nutrient-use efficiency, and improving stress tolerance [11, 36, 40, 46, 48, 150, 214]. Applications leveraging the seed microbiota include bioinoculants, microbiome engineering, and selective breeding for desirable microbiome traits to optimize plant–microbe interactions. Such strategies could facilitate the transition of conventional agriculture toward ecologically sustainable systems, minimizing environmental impact while maintaining crop productivity [32, 79]. Despite these promising advances, translating seed-applied bioinoculants from controlled trials to broad agricultural use faces significant hurdles. Key challenges include scaling up consistent production, navigating complex regulatory frameworks, and fostering farmer acceptance amid variable field performance. Addressing these barriers will be critical for fully realizing the benefits of the seed microbiota in sustainable cropping systems. Moreover, the complexity of plant–microbe interactions at the seed level presents both opportunities and challenges for research and practical applications. Future investigations should focus on addressing the heterogeneity of the seed microbiota, improving methodologies for microbiome manipulation, and establishing frameworks for integrating microbial applications into agricultural practices. Resolving these research gaps will strengthen the foundation for utilizing the seed microbiota to advance sustainable agriculture.

Seed-isolated bioinoculants, which include beneficial microorganisms applied to seeds prior to planting, represent a promising approach for enhancing plant growth, nutrient acquisition, and disease resistance. Bioinoculants establish beneficial microbial communities that persist throughout the plant life cycle by introducing these microorganisms at the earliest stages of plant development. This methodology reduces reliance on synthetic fertilizers and pesticides, thereby addressing the critical requirements for sustainable agriculture, especially in stages of development in which other treatments could be more aggressive and unspecific. This targeted stage has already been proven for seed-isolated microorganisms via the use of seed coatings, liquid formulations or long-term dispersal carriers [139, 165, 172, 173, 207]. In this sense, we have already conducted field tests in which the seed microbiota has been tested in maize via spray inoculation in plant-early stages, which has a positive effect on stress and a notable improvement in yield production. This is the case for the inoculation of the seed-borne strain Pseudomonas fulva, which has been proven to be effective in maize fields under water scarcity conditions [104]. Moreover, as coinoculants for legumes together with conventional Rhizobia inoculants, a series of studies have demonstrated the efficiency of using inoculants based on seed-isolated strains [37, 169, 198]. Strategies such as crop rotation, organic amendments, and minimal tillage support the establishment and maintenance of beneficial microbial communities, thereby creating a synergistic effect with seed-applied bioinoculants [199].

On the other hand, microbiome engineering represents a transformative approach for optimizing the seed microbiota to achieve targeted agricultural outcomes. This technique offers a pathway for improving plant growth, disease resistance, and stress resistance through selective enrichment of specific microbial taxa or engineering of strains with enhanced capabilities [200]. In addition to natural selection, synthetic microbial consortia represent another frontier in microbiome engineering. These consortia combine complementary microbial species, such as nitrogen fixers, phosphorus solubilizers and biocontrol strains, to create synergistic effects [117, 194]. Researchers have developed synthetic community inoculants capable of providing heightened protection against multiple pathogens by increasing the production of antimicrobial compounds and their coordination [97, 117, 118]. Crop breeding to increase microbial compatibility focuses on the selection of plant traits that promote beneficial seed microbiota [98, 179]. This strategy leverages natural interactions between plants and microbes to develop cultivars that recruit and sustain advantageous microbial communities [83, 179, 180]. For example, the introduction of Paraburkholderia phytofirmans into the flowers of wheat induced changes in the microbial community associated with the plant, and consequently, effects on plant growth and development were observed [147]. Breeding programs that incorporate microbiome-supportive traits, such as the increased production of signaling compounds or secondary metabolites, may provide a pathway for developing plants with optimized seed microbiota. This approach not only supports immediate crop productivity but also contributes to the long-term sustainability of agricultural systems by promoting resilient and diverse microbial communities [10, 41, 48, 99, 205]. These examples illustrate how the application of the seed microbiota can serve as a cornerstone for sustainable agriculture by balancing productivity with environmental stewardship.

However, translating seed‐isolated bioinoculants from the laboratory and small-scale trials to widespread agricultural use remains challenging. Large-scale production often results in batch-to-batch variability and reduced microbial viability, undermining product consistency in the field (phenotypic heterogeneity within inoculant populations) [217]. Similarly, formulation stability is constrained by limited shelf-life—solid carriers typically sustain viability for only 8–12 months, whereas liquid formulations rarely exceed 24 months without stringent temperature control [218]. Compounding these issues, regulatory frameworks for biofertilizers and biostimulants are highly fragmented across regions; in the EU, divergent national rules replace a unified standard, and in the USA, clear guidelines for microbial products remain lacking, leading to lengthy and costly registration processes that favor large firms over smaller innovators [219]. In the absence of predictable performance, farmer adoption stalls—field trials often yield inconsistent benefits under diverse soil and climate conditions, eroding grower confidence, whereas economic uncertainties and variable quality standards discourage investment by both smallholders and large producers [220]. Finally, compatibility issues with existing agrochemicals and tillage practices can negate inoculant efficacy unless tailored integration protocols and robust extension services are established to ensure synergistic outcomes [221].

## Discussion and conclusions

Seed microbiota research has emerged as a transformative field, providing novel insights into plant–microbe interactions and their significant implications for agriculture [7, 8]. This microbiota is largely relevant in many crops, and its relevance is much more common than it was a decade ago, indicating its relevance even in seagrass seeds [10, 12, 26, 97, 125, 222, 223]. Seed-associated microbes influence plant health through their roles in nutrient acquisition, pathogen defense, stress tolerance, and overall plant fitness [45, 127, 136, 138]. This microbial community is shaped by a complex interplay of factors, including host genetics, vertical and horizontal transmission mechanisms, environmental contexts, and microbial interactions within seeds [36, 47, 96, 153, 179, 215, 223, 224]. Although substantial progress has been made, numerous challenges and opportunities remain in elucidating the potential of the use of the seed microbiota to increase agricultural sustainability.

Building on our understanding of the significance of the seed microbiota, exploring how microbial inheritance, especially through vertical transmission, underpins these interactions in establishing a stable microbial community across generations is crucial [17, 21, 40, 127, 135, 162, 179]. Studies have demonstrated that maternally inherited microbes increase germination and seedling vigor by promoting nutrient acquisition and pathogen resistance [11, 14, 110, 225]. Horizontal transmission complements this inheritance by introducing diversity, enabling the seeds to adapt to specific environmental conditions. Studies on cereal crops and legumes have demonstrated that seeds exposed to diverse soil microbiota contain beneficial microbes, thereby increasing stress resistance and nutrient cycling [7, 40, 48, 110, 150, 200]. The duality of microbial transmission pathways highlights the adaptive potential of the seed microbiota. Plant-driven selection mechanisms further refine microbial assembly and selectively recruit microbes that align with the physiological and ecological requirements of plants. These selective pressures underscore the intricate co-evolution of plants and their microbiota, resulting in specialized interactions that increase plant resilience [10, 31, 78, 80, 132, 133, 147, 176, 181]. In this sense, the application of the seed microbiota in sustainable agriculture requires a new ecosystem perspective if we aim to harness all their potential. Considering one-for-all solutions as conventional bioinoculants that are currently commercialized would be an error considering the pre-adapted and specific characteristics of this microbiota. This does not mean that they cannot have a wide application spectrum, but we cannot keep dispersed perspectives for such promising solutions. However, ensuring the stability and scalability of these bioinoculants across diverse agricultural systems remains a challenge, and more studies are needed to understand their mechanisms and potentialities [11, 81, 110, 148].

Future research must address key knowledge gaps to advance this field. The temporal dynamics of the seed microbiota, particularly during dormancy and germination, require further exploration to identify persistent and transient taxa and their functional roles [19, 81, 82, 115, 134, 226, 227]. Longitudinal studies across different crops and environments could provide insights into microbial succession and its implications for plant development [152, 153, 201]. Additionally, the molecular and genetic underpinnings of plant-driven selection processes merit investigation to inform breeding programs aimed at optimizing microbial interactions [47, 153, 178, 215, 224]. Emerging technologies, such as metagenomics, transcriptomics, and synthetic biology, hold promise for overcoming current limitations [32, 79, 93, 97]. Precision editing of microbial genomes, enabled by CRISPR-Cas systems, can enhance the beneficial traits of seed-associated microbes, thereby creating bioinoculants with tailored functionalities. However, the ecological risks associated with introducing engineered microbes into agricultural systems should be carefully managed via robust biosafety protocols and long-term monitoring frameworks [228–230].

The sustainable adoption of seed microbiota-based innovations necessitates a holistic approach that considers the ecological, economic, and social dimensions [30, 95]. Farmer education and outreach programs are essential for promoting an understanding of microbial technologies and their benefits. Furthermore, policy frameworks must balance innovation with environmental stewardship to ensure that the introduction of non-native or engineered microbes does not disrupt local ecosystems [11, 31, 104]. Collaborative efforts among researchers, policymakers, and agricultural stakeholders are critical for achieving this balance.

In conclusion, the seed microbiota represents a powerful tool for transforming agricultural practices in an era of growing environmental challenges. By leveraging the functional potential of seed-associated microbes, it is possible to reduce reliance on chemical inputs, increase crop resilience to abiotic and biotic stresses, and promote the sustainable intensification of agriculture. Continued interdisciplinary research coupled with practical applications will pave the way for scalable and environmentally friendly solutions that address the dual imperatives of food security and ecological sustainability. As the gap between fundamental science and field applications is bridged, the seed microbiota is poised to play a central role in shaping the future of global agriculture.

## Data Availability

Not applicable.
